# Maintenance of peri-implant health in general dental practice

**DOI:** 10.1038/s41415-024-7406-8

**Published:** 2024-05-24

**Authors:** ﻿Jeniffer Perussolo, Nikolaos Donos

**Affiliations:** Centre for Oral Clinical Research, Institute of Dentistry, Faculty of Medicine and Dentistry, Queen Mary University of London, Turner Street, London, E1 2AD, UK

## Abstract

The long-term maintenance or restoration of peri-implant tissues‘ health depends on the strategic implementation of preventive measures and interventions. These measures should be initiated before implant placement and continued throughout a patient's lifetime, as part of a tailored and comprehensive supportive peri-implant care (SPIC) programme. Central to the clinical efforts of maintaining and rehabilitating peri-implant tissues are several key factors, including the ongoing assessment and frequent monitoring of tissue health and stability, proactive oral health promotion, the control of risk factors and indicators and the provision of professional plaque biofilm removal. It is of paramount importance to underline that SPIC should not limit its scope exclusively to patients already in a state of peri-implant health; in fact, it is imperative that it should extend its protective effect to individuals who have been previously diagnosed and treated for peri-implant diseases, focusing on preventing its recurrence and progression, thereby avoiding further complications, such as implant loss.

This narrative review presents an overview of the current literature on the maintenance of peri-implant tissues‘ health and the steps of SPIC providing insights into the critical factors to be considered when managing dental implant patients in the general dental practice.

## Key points

- Provides an overview of the current literature on the maintenance of peri-implant tissues‘ health.
- Presents the steps of supportive peri-implant care.
- Discusses critical factors to be considered when managing dental implant patients in general dental practice.

## Introduction

Dental implants are a reliable treatment option for rehabilitating complete or partial edentulous patients.^1^^,^^2^ Nevertheless, complications may occur at different time points, from early implant failures to later stages which are mainly associated to biological complications, or in some instances,^3^^,^^4^ due to mechanical failure, including implant or screw fracture, screw loosening and suprastructure deformity.^3^^,^^5^

Biological complications encompass the two most common problems following implant placement, namely peri-implant mucositis and peri-implantitis.^6^ Peri-implant mucositis manifests as inflammation of the soft tissues around the implant without associated bone loss.^7^ It is a clinically manageable condition; however, when neglected, may progress to peri-implantitis, a more severe problem characterised by both inflammation and progressive bone loss.^8^ Approximately 45% and 20% of implant patients may present peri-implant mucositis or peri-implantitis, respectively,^9^^,^^10^ while implant loss may range from 0-14% at patient level.^9^ For most cases, plaque accumulation is the primary aetiological agent,^6^^,^^8^ while factors such as past diagnosis of periodontitis, suboptimal plaque management, and the absence of regular supportive peri-implant care (SPIC), may enhance the risk for peri-implantitis.^8^

Considering the high prevalence of peri-implant diseases,^9^^,^^10^ any patient rehabilitated with dental implants might eventually encounter these complications. Hence, the successful, long-term maintenance and/or restoration of peri-implant tissue health depends on the provision of appropriate preventive measures and interventions, which include: i) assessment and monitoring of the peri-implant tissue condition; ii) oral/peri-implant health promotion, behavioural change, and controlling of other risks and management of systemic diseases (ie diabetes); iii) periodical professionally delivered plaque removal as part of an SPIC, including elimination of plaque-retentive factors; and iv) treatment of peri-implant disease.^11^^,^^12^

Implementing a consistent SPIC not only contributes to the maintenance of peri-implant tissue health^13^ but also has the potential to prevent or delay the onset of peri-implant diseases, particularly among high-risk groups.^14^ General dental practitioners (GDPs) play a fundamental role in providing SPIC. Nevertheless, research indicates a lack of confidence and knowledge gap among dental professionals diagnosing and managing the early stages of peri-implant diseases. In the UK, approximately 13% of GDPs report avoiding probing around implants for fear of complications or potential medico-legal consequences, while 14% do not assess implants at all.^15^

This narrative review aims to summarise the existing literature on the maintenance of peri-implant tissues‘ health and the steps of SPIC, providing the relevant evidence on the critical factors to be considered when managing dental implant patients in the general dental practice.

## The starting point

**Fig. 1:**
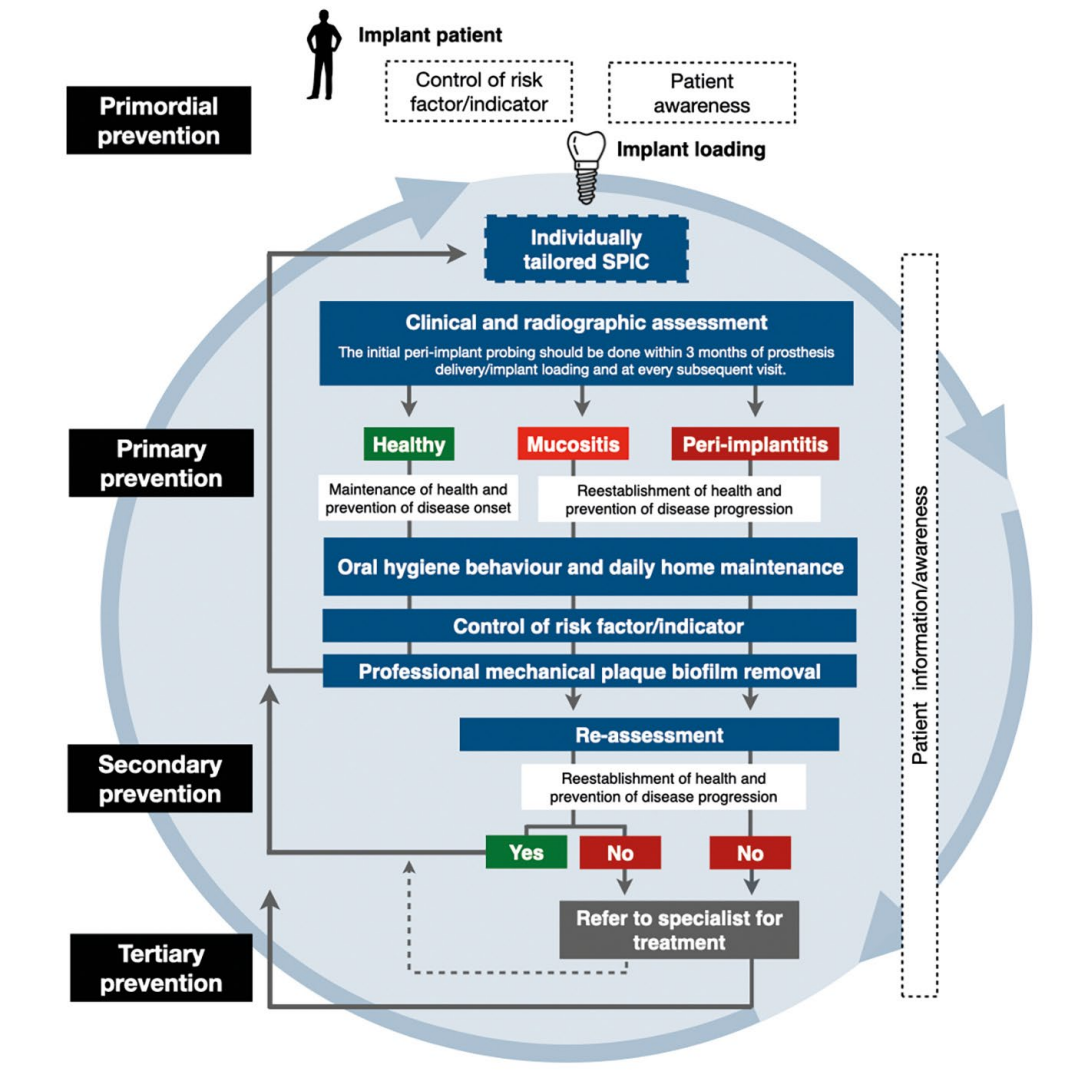
This flowchart illustrates the various stages involved in maintaining or restoring the health of peri-implant tissues, based on the established diagnosis and the implementation of suitable preventive measures and interventions. Primordial prevention = prevention before and during implant placement/loading; primary prevention = prevention of disease onset once the implants are loaded and in function; secondary and tertiary prevention = management, prevention of recurrence and progression of disease in sites diagnosed and treated for peri-implant diseases

Recently, the European Federation of Periodontology (EFP) has published the S3-level clinical practice guideline on the Prevention and treatment of peri-implant diseases,^11^ stating that the prevention should begin during dental implant planning and continue through the surgical placement and prosthetic loading (primordial prevention).^11^^,^^16^ At this stage, prevention is focused on advising patients and controlling any risk factor/indicator (eg poor plaque control, unstable periodontitis and glycaemic level) of peri-implant disease. Once the implants are loaded, an individually tailored SPIC programme should be planned, including periodical evaluation of peri-implant tissues and control of known risk factors by self-administered and professional plaque biofilm removal (primary prevention).^11^^,^^16^ These steps should target both patients with peri-implant health and also those with a diagnosis and previous treatment of peri-implant diseases. In these situations, prevention of recurrence and progression of disease to avoid implant loss is an essential requirement (secondary and tertiary prevention)^11^ (Fig. 1). GDP involvement during all treatment phases, including early peri-implant disease detection and specialist referral for advanced care, is key in prevention.

### Patient awareness

It has been suggested that patients seeking rehabilitation with dental implants present with high expectations and the unrealistic perception that dental implants are a worry-free treatment not requiring as much care as the natural teeth.^17^ Therefore, it is part of the primary duty of the dentist/dental hygienist/therapist to effectively educate and motivate patients regarding the significance of SPIC and home care of their dental implants.^18^

Furthermore, patients should be informed about the possible complications and the consequences of progression of peri-implant mucositis to peri-implantitis, as well as clinical signs and symptoms (ie pain, bleeding, suppuration) that should prompt them to contact their dentist/dental hygienist/therapist.^6^ A recent study has shown that only 32% of patients reported receiving guidance on managing bleeding around dental implants. Among them, 33% were advised to contact their dentist, while 28.6% received recommendation to use a mouthrinse and to improve their oral hygiene (OH) practices. The remaining 33% were not able to specify the recommendations given.^18^ This brings to attention a potential underestimation from the dental team of the risks associated to a long-lasting inflammation.^7^^,^^19^

It would be advantageous if the above-mentioned information is provided to patient as an information booklet on dental implant care and peri-implant diseases.^17^

## Supportive peri-implant care

The SPIC has been termed in previous studies as supportive care, supportive therapy, supportive peri-implant therapy, peri-implant maintenance therapy, maintenance therapy and supportive peri-implant maintenance, among others.^11^^,^^12^^,^^20^

The tailored follow-up programme includes four essential steps in each SPIC appointment: monitoring peri-implant tissue health, fostering oral health through motivation and risk management, professional plaque removal, and treating infected sites.^11^^,^^12^

### Clinical and radiographic monitoring of peri-implant tissues

The clinical and radiographic evaluation of the peri-implant tissues should include: i) visual inspection of the peri-implant mucosa to identify potential signs of inflammation; ii) assessment of bleeding on probing (BOP) and/or suppuration; and iii) changes in probing depth (PD), mucosal margin level and marginal bone level (MBL). Sulcus bleeding index may be considered where probing is not feasible.^11^^,^^21^ The width of peri-implant keratinised mucosa (KM) should also be recorded, whenever possible.^14^ The initial (baseline) peri-implant probing should be done either at implant loading^22^ or within the first three months after crown delivery^11^ and at every subsequent clinical examination, ideally at six sites per implant, using a periodontal probe and a light probing force.^11^

**Fig. 2:**
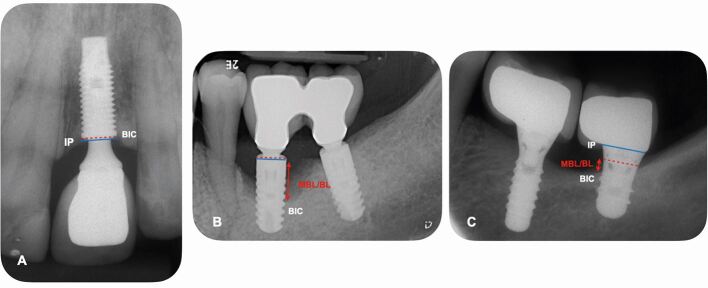
Periapical radiographs showing the MBL/BL (marginal bone level/bone loss) around dental implants. a, b, c) Measured as the distance from the most coronal point of the intraosseous segment (dotted red line) of implant or implant platform (IP) (blue line) to the deepest bone-to-implant contact (BIC). c) The tissue level implants are placed with the rough surface of the implant up to the crestal bone with the smooth neck in contact with soft tissues. Therefore, any bone loss occurring up to smooth-rough surface interface (dotted red line) can be considered physiological remodelling, while bone loss occurring apical to that point can be considered pathological

Additionally, a baseline intra-oral periapical radiograph at implant loading^23^ or at the completion of biological bone remodelling^11^ should be obtained to document MBL, measured as the distance from the most coronal point of the intraosseous segment of the implant to the point where bone first contacts the implant surface, as shown in Fig. 2. At subsequent visits, taking an intra-oral radiograph to assess the MBL changes over time is recommended if after clinical evaluation an increase in PD in combination with BOP/suppuration is identified or in case a mechanical complication (ie screw loosening or fracture) is suspected.^23^

Considering that plaque biofilm is the main aetiological cause of peri-implant disease and history of periodontal disease increases the chances for peri-implantitis, full-mouth plaque score should also be recorded together with the full-mouth routine periodontal examination. Plaque can be detected and measured objectively with the aid of a periodontal probe or disclosing agents.

In addition, a recent study has demonstrated that about 70% of implants exhibiting KM <2 mm and diagnosed with peri-implant diseases presented some level of brushing discomfort (BD) during supervised OH.^24^^,^^25^ Therefore, when BD is reported, thorough assessment of the peri-implant tissues must be considered.^24^^,^^25^

The information collected during examination is essential for establishing the health/disease status of peri-implant tissues. The 2017 American Academy of Periodontology/EFP World Workshop on the Classification of Periodontal and Peri-Implant Diseases and Conditions set diagnostic guidance for peri-implant disease (Table 1),^6^^,^^26^ which has been recently updated as part of the EFP S3-level clinical practice guideline.^11^

**Table 1:** Case definitions in day-to-day clinical practice for peri-implant health, peri-implant mucositis and peri-implantitis

	Clinical and radiographic parameters		
Case definition	BOP and/or suppuration*	Increased PD**	Bone loss^†^
Peri-implant health	No or presence of a single bleeding spot^11^	No	No
Peri-implant mucositis	Yes (more than one bleeding spot or profuse bleeding)^11^	Yes	No
Peri-implantitis	Yes	Yes	Yes
Key: * = Suppuration will occur more frequently in implants with ‘progressive' bone loss. ** = Increased PD compared to previous evaluation or PD ≥6 mm (if data on previous examination of implant not available). ^†^ = Bone loss beyond bone level changes resulting from initial bone remodelling or bone levels ≥3 mm apical to the most coronal portion of the intraosseous part of the implant (if data on previous examination of implant not available).			

It is important to emphasise that it is challenging to determine a range of PD compatible with peri-implant health, as dimensions of mucosa may vary due to implant position (eg deeply placed implants).^6^^,^^27^ In addition, the amount of initial physiological bone remodelling may vary due to different types of implant designs, surface features, surgical and loading protocols. These are the reasons why clinicians should obtain baseline radiographic and probing measurements after implant loading so changes in the peri-implant tissues can be monitored over time.

Table 2 presents a checklist with parameters to be considered during SPIC appointments.

**Table 2:** Checklist of main clinical and radiographic parameters to be considered during SPIC appointment in a general dental practice

Assessment and monitoring of peri-implant tissues condition	
Procedures	Checked
**Clinical assessment**	
Visual inspection of peri-implant mucosa Identify potential signs of inflammation, such as swelling, redness, tenderness and spontaneous bleeding	☐
BOP and/or suppuration Recorded by the presence or absence of bleeding/suppuration after 15 s of gentle probing	☐
Probing depth* Measured (mm) ideally at six sites per implant, from the peri-implant mucosa margin to the bottom of the peri-implant sulcus/pocket	☐
Peri-implant mucosal margin level Measured (mm) from the abutment/fixture junction to the peri-implant mucosal margin	☐
Peri-implant keratinised mucosal width Measured (mm) as the distance from the peri-implant mucosal margin to the mucogingival junction at the mid-buccal aspect of each implant^25^	☐
Presence of pain/discomfort in the area of the implant Recorded as presence or absence of pain/discomfort or with the aid of a questionnaire/pain assessment scale ie visual analogue scale^25^	☐
Full-mouth plaque score Score the presence or absence of plaque in every tooth and implant	☐
Full mouth routine periodontal examination Assess PD, BOP, clinical attachment level and gingival recession. Also evaluate access for OH	☐
**Radiographic assessment**	
MBL** Measured (mm) as the distance from the most coronal point of the intraosseous segment of the implant to the deepest bone-to-implant contact	☐
Implant-supported restoration assessment (ie correct fit of implant components and the suprastructure, excess of cement, overcontour)	☐
Key: * = The initial (baseline) peri-implant probing should be done either at implant loading^21^ or within the first three months after crown delivery^11^ and at every subsequent clinical examination. ** = A intra-oral periapical radiograph should be obtained at implant loading^22^ or at the completion of physiological bone remodelling^11^ and at subsequent visits if after clinical evaluation an increase in PD in conjunction with BOP/suppuration is identified or in case a technical/mechanical complication.^22^	

### Oral hygiene behaviour and daily home maintenance

The role of dental plaque biofilm on the onset of inflammatory response and progression of MBL around dental implants has been broadly described.^7^^,^^28^^,^^29^^,^^30^^,^^31^^,^^32^^,^^33^ Hence, patient self-performed plaque control is one of key factors for prevention of development and treatment of peri-implant diseases.^11^^,^^16^^,^^34^

A recent study found that while over 90% of participants learned to clean their dental implants, only 40% actually practised it under the supervision of a dental professional.^18^ Knowledge gaps in home and professional care persist, with some fearing self-care may damage implants.^17^ These results highlight the importance of assessing patients' OH procedures at the dental practice to identify factors which may jeopardise adequate plaque control, such as inadequate access to OH and presence of plaque-retentive factors. Besides instructing patients periodically, it is important to demonstrate the use of different devices to overcome any specific challenges.^11^ Also, patients should be questioned about presence of pain/discomfort during OH of dental implants and dentine hypersensitivity in the adjacent teeth, as these factors may hamper OH. For patients with disabilities or additional needs, caregivers should also be educated on the significance of maintaining optimal OH and provided with detailed instructions for assisting OH.

**Fig. 3:**
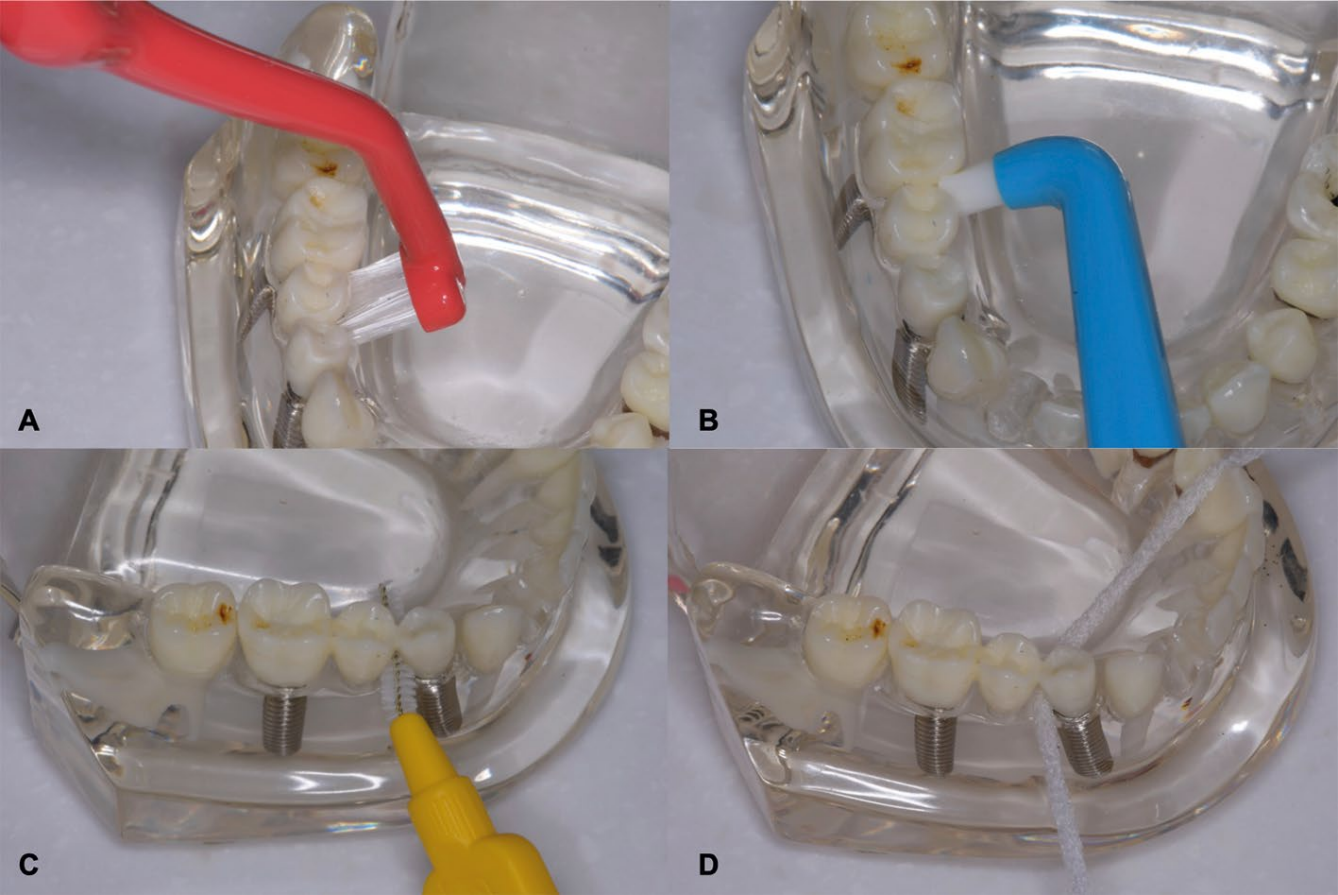
Example of OH devices for dental implant care. a) Angled neck implant toothbrush which provides improved access to difficult to reach areas. b) Single-tuft brush. c) Interdental brush. d) Spongy floss with a stiffened end

Currently, a multitude of devices/materials are offered for OH purpose (Fig. 3), including toothbrushes (manual, counter-rotational powered, sonic, single-tuft brushes), interdental brushes, dental floss, toothpaste, oral irrigators and mouthrinses,^35^^,^^36^^,^^37^^,^^38^^,^^39^ which should be instructed and used according to patient needs.

#### Toothbrush

Based on current data, there is still no specific standard of care for OH procedures for dental implants and it remains inconclusive which type of toothbrush (eg manual or powered toothbrush), frequency and duration of toothbrushing is most effective in maintaining peri-implant health and reducing the risk of disease recurrence.^11^ Nevertheless, a counter-rotational powered toothbrush has demonstrated better results in terms of tissue inflammation resolution and implant survival compared with manual toothbrushing.^40^ Notably, powered brushes improved plaque control in less accessible interproximal sites compared to manual cleaning of more accessible (buccal and lingual) sites.^39^ Patients who are not adept with a manual toothbrush or individuals with disabilities may particularly benefit from using an electric toothbrush to improve plaque removal.^41^ When manual and sonic toothbrushes were compared, no difference was identified, with both toothbrushes maintaining peri-implant tissue health over a one-year period.^42^ Regarding the frequency of toothbrushing, no influence on PD, MBL and BOP was observed.^43^

Irrespective of the frequency and type of toothbrush used, patients' education in OH is essential to maintain peri-implant tissues‘ health and to prevent the onset of peri-implant diseases. Therefore, particularly for peri-implantitis-treated patients, customised regimens with twice-daily brushing of implants and teeth, using either powered or manual toothbrush, are recommended.^11^

#### Interproximal cleaning

To date, conventional manual methods for interproximal plaque removal (such as dental floss, interproximal brushes [IB]) remain the clinical standard of care.^44^

Dental floss should be carefully used, particularly around implants with exposed threads and misfit, to prevent deposition of floss fibres on the implant surfaces, which could lead to plaque-related peri-implant inflammation and bone loss.^45^ In addition, the growing interest in finding devices that are capable of overcoming the challenges of cleaning implant-supported restorations has driven the creation of alternative devices for interproximal cleaning. A ‘waist-shaped' IB resulted in significantly lower plaque scores than a straight IB, mainly due to the higher cleansing effect on the buccal and lingual surface angles.^35^

Oral irrigators, also called dental water jets or water flossers, are electric devices that deliver pulsating fluid under controlled pressure and are particularly beneficial around implants with limited access for OH (ie implant-supported overdentures, patients with decreased manual dexterity).^38^ Oral irrigators may result in greater bleeding reduction (81.8% than string floss (33.3%).^46^ When used as an adjunct to twice-daily brushing, they result in improved clinical and biochemical outcomes compared to IB and manual brushing alone.^38^ Adding antimicrobial agents instead of water, such as 0.06% chlorhexidine (CHX), has shown to enhance oral irrigation's efficacy, reducing inflammation and improving peri-implant mucositis severity.^37^ However, some users may experience pain and difficulty with oral irrigators use.^35^

Advising patients on daily interproximal cleaning is essential for long-term peri-implant health. In patients with peri-implant mucositis, in addition to regular OH, the use of oral irrigator with water may also be considered.^11^

#### Self-administered antiseptics

Treatment with mechanical plaque removal, OH reinforcement and regular homecare use of a 0.12% CHX mouth rinse, two times a day during two weeks, was effective in decreasing mucositis, but did not completely resolve inflammation.^47^ The daily use of a 0.03% CHX and 0.05% cetylpyridinium chloride (CPC) mouthwash as an adjunct to mechanical plaque removal demonstrated benefits in reducing BOP and the total bacterial count around dental implants. However, the proportion of peri-implant mucositis resolution was similar among patients rinsing or not rinsing (58.3% and 50%, respectively).^39^ One of the disadvantages of the use 0.03% CHX and 0.05% CPC mouthrinse was the higher levels of staining on the teeth or tongue.^48^ Some patients may also experience a burning sensation of oral mucosa and change in taste.^48^ In patients with peri-implant mucositis, the domiciliary use of a CHX gel daily for a period of two weeks has also shown to reduce PD after six months.^49^

Previously, the chemical plaque control by oral rinses has been considered to present limited additional benefit to patient-administered mechanical plaque removal.^34^ However, the S3-level guidelines suggested that professionally guided self-performed administration of antiseptic mouthwashes (ie CHX and herbal-based) adjunctive to professional mechanical plaque removal (PMPR) may be beneficial, particularly for peri-implant mucositis patients.^11^

### Control of risk factors and indicators

The identification, control and monitoring of risk factors/indicators related with the onset and progression of peri-implant diseases is crucial.^11^ Strong evidence supports the history of periodontitis, inadequate plaque control and irregular SPIC as risk factors for peri-implantitis development.^6^^,^^8^^,^^50^ Prior studies have demonstrated that individuals susceptible or presenting with periodontitis were at a higher risk for peri-implantitis.^19^^,^^32^^,^^51^^,^^52^^,^^53^Therefore, it is important to prioritise not only the maintenance of dental implants but also the assessment and care of periodontal tissues, while also educating patients about the significance of preserving the periodontal stability achieved post-periodontal treatment and the associated risks to periodontal disease relapse.^23^

Various studies have suggested that the chronic hyperglycaemia observed in diabetes mellitus (DM) patients may lead to a persistent local and systemic inflammatory response.^54^^,^^55^^,^^56^ Higher rates of peri-implantitis and bone loss were observed over time in patients with uncontrolled DM compared with those showing good glycaemic control.^11^^,^^16^^,^^57^ Nonetheless, it is still inconclusive if diabetes increases the risk of peri-implant diseases.^8^ Irrespective of that, DM patients should be advised on the potential risk for development of peri-implant diseases and importance of glycaemic control before, during and after treatment.^11^

Smoking has also been shown to present a negative impact on peri-implant tissues, leading to a higher occurrence of peri-implant mucositis (48.6% versus 43.9%) and peri-implantitis (30.5% versus 19.7%) in current smokers compared with former smokers.^58^ A clear relationship between smoking duration and elevated peri-implantitis risk has also been found.^58^Despite limited evidence on the impact of smoking, including e-cigarettes or water pipe, on peri-implant tissues, patients should be informed of its potential risks and harmful consequences.^11^ The implementation of validated smoking cessation interventions and relevant advice to of all smokers is recommended.

**Fig. 4:**
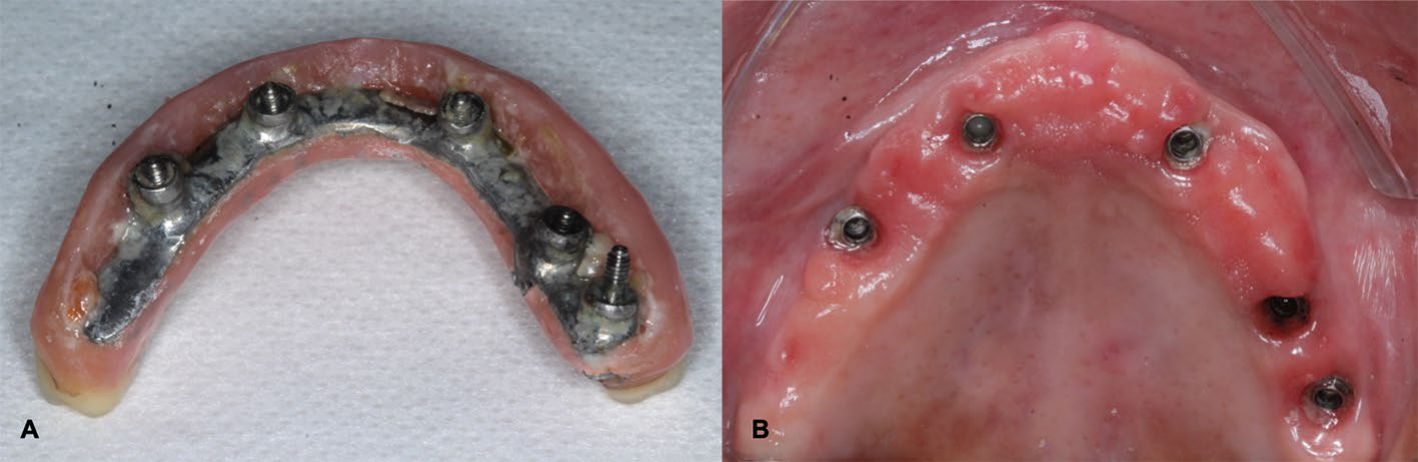
a, b) Full-arch maxillary implant-supported fixed restoration with a poor buccal contour and inadequate access to OH leading to plaque accumulation and soft tissue inflammation

In addition, other local factors, such as presence of submucosal cement and restorations that jeopardise access to OH, have also shown to negatively influence the health of peri-implant tissues.^8^ In everyday clinical practice, it is not uncommon to come across implants placed under less-than-optimal conditions, and with prosthetic solutions leading to restorations with inadequate access to OH (Fig. 4). Most implants with peri-implantitis had compromised cleaning due to access difficulties.^59^^,^^60^ Hence, prosthetic reconstructions require an emergence profile that prevents plaque build-up and tissue inflammation.^61^ If needed, prostheses may be removed for cleaning and adjustment ensuring proper access for OH and peri-implant assessment.^11^

**Fig. 5:**
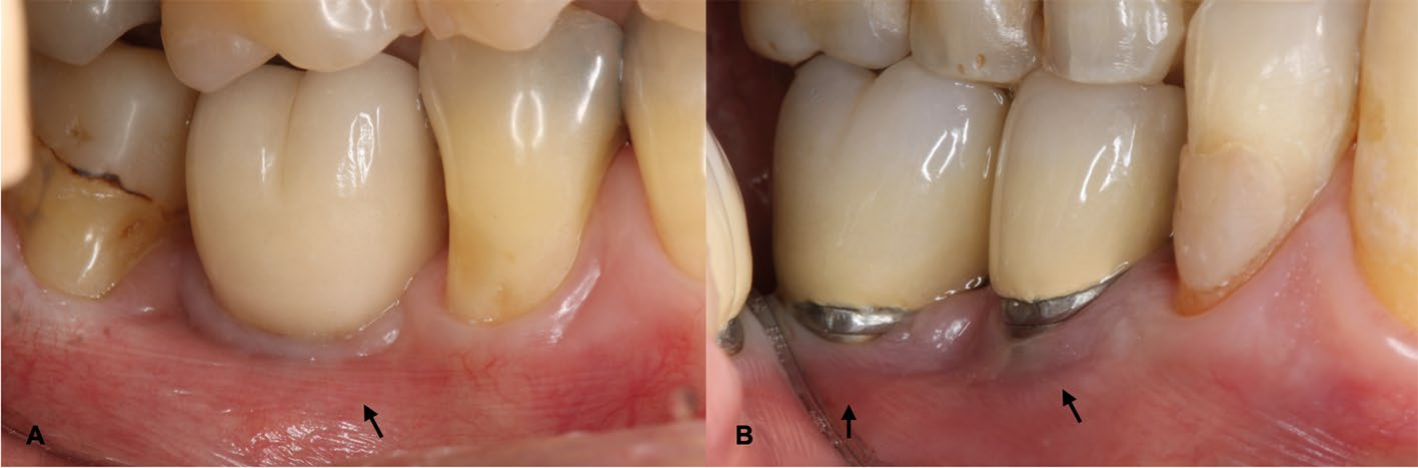
a, b) Dental implants in the posterior region of the mandible exhibiting inadequate KM width (ie <2 mm)

Inadequate KM (<2 mm) (Fig. 5) width has been associated with greater soft tissue inflammation, mucosal recession^25^^,^^62^^,^^63^^,^^64^^,^^65^^,^^66^^,^^67^^,^^68^^,^^69^ and marginal bone loss around implants.^25^^,^^69^^,^^70^ Hence, in patients with dental implants exhibiting insufficient KM width and BD, recurrent inflammation, or increased recession of the peri-implant mucosa, KM augmentation may be considered.^11^^,^^71^ Risks and benefits should be weighted and any potential factors contributing to inflammation and BD should be identified and eliminated.^24^ While the association between increased peri-implant mucosal thickness and prevention of peri-implant diseases lacks evidence,^11^ thicker soft tissues are associated with improved aesthetics and reduced mucosal recession at implant sites.^72^

### Professionally administered plaque biofilm removal

**Fig. 6:**
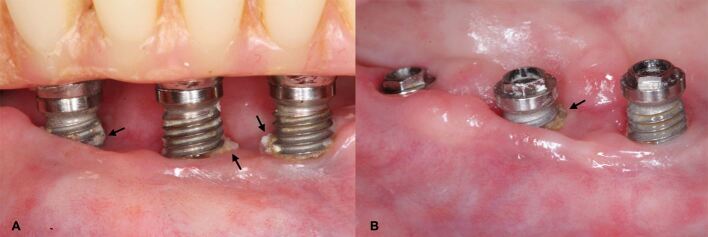
a, b) Dental implants with exposed thread surfaces which promoted plaque accumulation and calculus formation (indicated by arrows)

Besides providing an individualised oral healthcare plan, PMPR of the implant/s and dentition should also be performed. PMPR around implants involves the removal of supramarginal and submarginal hard and soft deposits from the dental implant surface and/or its suprastructure without damaging both surfaces by using a selection of hand or powered instruments and/or polishing tools (Fig. 6).^34^ In mucositis and peri-implantitis, non-surgical periodontal treatment (NSPT) should be provided as an essential step for resolving inflammation. Depending on the response of peri-implant tissues, additional NSPT or surgical treatment (peri-implantitis) is recommended.

In the literature, a range of instruments have been described to be used during SPIC. Frequently, SPIC is performed with a combination of hand (ie titanium, Teflon, carbon fibre, plastic curettes) and mechanical/powered-driven instruments, such as sonic/ultrasonic devices, abrasive air-polishing systems and lasers with or without adjunctive antimicrobial therapy (ie irrigation with CHX solution). The selection of instruments should also be guided by clinical judgement and based on the individual case requirements.

#### Hand and power-driven instruments

**Fig. 7:**
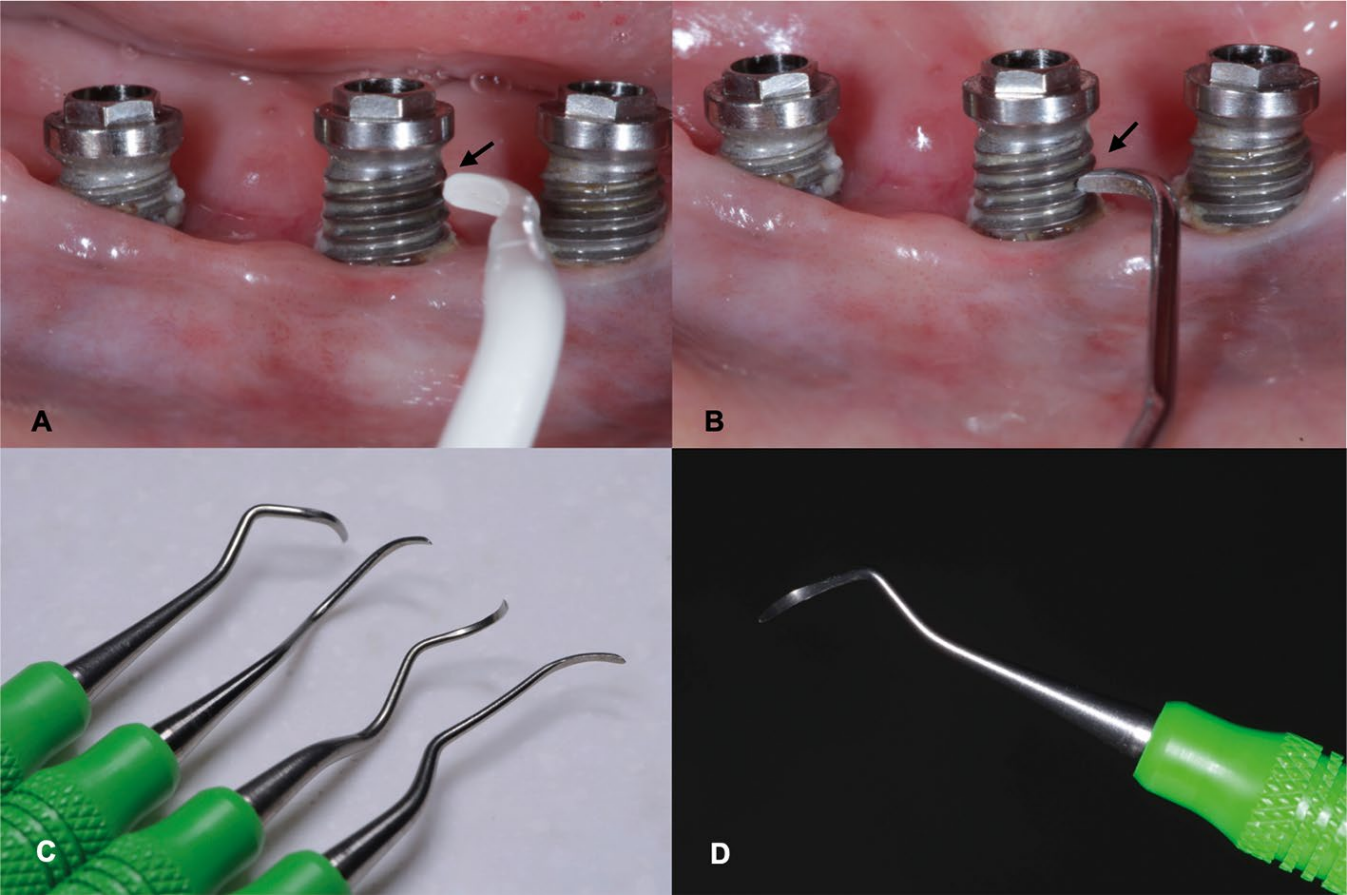
Manual instruments for non-surgical peri-implant treatment as part of supportive peri-implant care. Accessibility to plaque removal and the adaptation of manual and power-driven instruments to the implant surface can often be challenge. a) Plastic curette with a tip size that does not adapt/fit between implant threats. b) Conventional stainless steel curettes have smaller tips and adapt between implant threats but can leave severe damage on the implant surface and render it conducive to future plaque accumulation. c, d) Titanium curettes have a small tip and are strong enough to remove light to moderate calcified deposits on implants

Titanium and carbon fibre curettes can effectively remove mild-to-moderate calcified deposits on implant surfaces and may also lead to less surface damage compared to metal instruments.^73^^,^^74^In contrast, plastic curettes may lack the strength and size for such tasks. Conventional steel curettes can significantly ‘scratch' the implant surface, promoting plaque build-up. Hence, their use should be limited to calculus removal without contacting the implant surface (Fig. 7).^75^ Ultrasonic instruments with a variety of tips (eg plastic-coated, carbon fibre) may be used for removal of supramucosal and submucosal calculus and plaque biofilm deposits, and are more effective than hand-plastic curettes.^76^ Plaque biofilm may also be removed using rubber cups and polishing-paste or air-polishing devices.

**Fig. 8:**
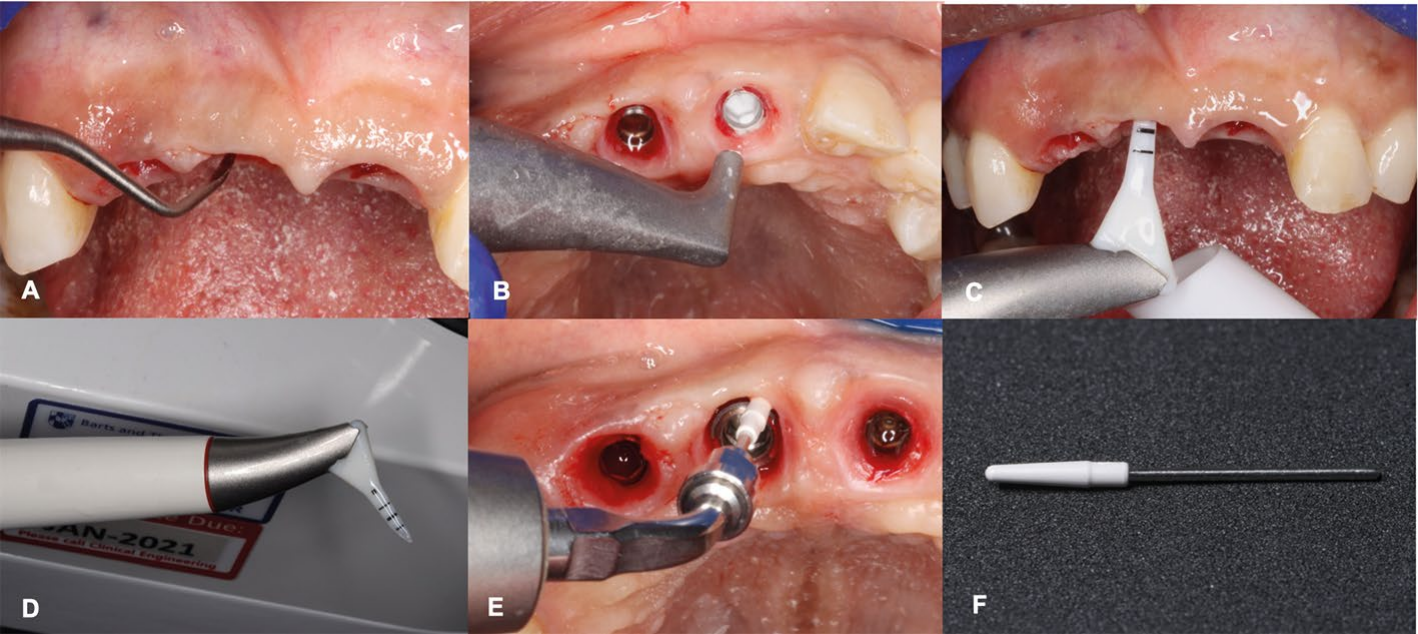
Non-surgical peri-implant treatment sequence employing a glycine-based air-abrasive system. a) Initial removal of biofilm and calculus deposits using a titanium curette, followed by b) removal of both supra and submucosal biofilm up to 4 mm using an air-polishing device that dispenses a glycine-based grain powder and water. c) For biofilm removal in pockets deeper than 4 mm, a fine plastic nozzle (d) is employed along with powder delivery. e) To ensure a thorough and safe cleaning of sensitive surfaces on implant abutments and restorations, a polyether ether ketone tip-coating (f) is attached to an ultrasonic unit for subgingival cleaning up to 3 mm

Air-polishing devices have been introduced as a safe and efficient alternative to remove supramucosal and submucosal biofilm on dental implants and natural teeth, suggesting that they produce less marked surface changes due to less abrasive characteristics of amino acid glycine particles.^77^^,^^78^ The design of the working tip allows the powder to flow vertically, accessing visually uncontrolled or hand-inaccessible areas (Fig. 8). Various air-polishing devices exist, but those using reduced air pressure to deliver a glycine-based powder and water have demonstrated reduction in plaque score, inflammation, and the number of pockets ≥4 mm within a 12-month treatment period.^77^

Various approaches within SPIC have also been investigated. A randomised controlled trial, comparing implant curettes with sonic scalers or air-polishing use on a three-monthly SPIC programme, showed that the different preventive approaches resulted in no significant change in BOP, thus demonstrating that these approaches are efficient options in maintaining peri-implant health in the short-term.^79^

Therefore, specific recommendations on the type of instrument, depending on each case, may apply. In patients with peri-implant mucositis, power-driven instruments (ie ultrasonic devices with plastic-coated tips, air-polishing tool using glycine powder, chitosan brushes) or manual instruments may be considered as a single mode of PMPR.^11^ The combined use of abrasive air-polishing systems or diode laser to conventional PMPR may not be justified due to increased costs and the possibility of complications.^11^

For the initial non-surgical treatment of peri-implantitis, the use of curettes and/or sonic/ultrasonic devices is recommended. While limited research supporting the efficacy of lasers, air-polishing or antimicrobial photodynamic therapy, either as adjunctive or PMPR alone, is available, there is limited evidence on the additional benefits following their use.^11^

In patients already treated for peri-implantitis and under SPIC, it is still not known which PMPR regime is most effective in decreasing the recurrence of disease. Despite that, the use of manual curettes, ultrasonic/sonic instruments and air-polishing devices alone or in combination may be considered for biofilm removal.^11^

#### Adjunctive use of local or systemic agents

Although professional administration of antibiotics (topical or systemic) or topical antiseptic agents, such CHX and hydrogen peroxide, have been used as adjuncts to PMPR, particularly in non-responding and recurrent sites, they have shown limited additional effects^80^^,^^81^^,^^82^ and were not found to improve the efficacy of non-surgical therapy in reducing clinical signs of inflammation in mucositis cases.^34^ Therefore, the local and systemic use of antibiotics in mucositis cases, as well as its systemic use during NSPT of peri-implantitis, is not recommended, due to the impact of its use on public health and antibiotic resistance.^11^

In addition, professional local administration of antimicrobial agents, such as antiseptics during non-surgical treatment of mucositis and peri-implantitis cases and as part of SPIC, is not generally recommended due to uncertain benefits.^11^ However, it is always important to consider each case individually bearing in mind other health conditions or risks.

The use of other adjunctive measures such as probiotic tablets containing *Lactobacillus reuteri* strains may also be considered when treating peri-implant mucositis. Probiotics can offer some benefits by potentially modulating oral microbiota and immune response and improving inflammation control for up to three months.^83^

#### Clinical endpoints

While the treatment of peri-implant diseases is not within the scope of this review, it is important to consider that non-surgical interventions often prove effective in addressing peri-implant mucositis but also present with limitations when dealing with most peri-implantitis cases. If, after the non-surgical peri-implantitis treatment, deep pockets (>5 mm) along with BOP at more than one site and/or suppuration are present, additional surgical measures may be necessary, requiring consultation with a specialist. Upon completion of treatment, SPIC should always be re-instituted (Fig. 1).^11^

For those cases treated for peri-implant mucositis, the end point is ≤1 point of BOP and absence of suppuration, evaluated at 2-3 months post-intervention.^11^ If the inflammation has been resolved, recall appointments for SPIC are recommended.^11^ If inflammation persists (≥2 BOP sites, ≥1 sites with profuse BOP, or presence of suppuration), re-treatment should be rendered.^11^ For peri-implantitis, the non-surgical and surgical treatment end points should be evaluated at 6-12 weeks and six months after the intervention, respectively. The endpoint after peri-implantitis treatment will be PD ≤5 mm, ≤1 point of BOP, no suppuration and absence of progressive bone loss.

## Recall intervals

The lack of SPIC represents a high risk for peri-implant disease development and implant loss, while full compliance with a recommended SPIC interval results in lower risk for biological complications.^84^^,^^85^ A previous systematic review determined that a minimum SPIC interval of 5-6 months should be considered to reduce chances of biological complications.^20^ The current EFP S3-level clinical practice guideline suggests an initial three-month SPIC interval post-treatment for peri-implantitis, followed by a 3-4-month interval during the first year. Thereafter, SPIC should be established on a case-by-case basis according to patient-, implant- and restoration-based risk profiling and needs (eg 3-, 6- or 12-month intervals).^11^

To facilitate selection/design of recall interval and to predict the risk of a patient/implant develop peri-implant diseases, the Implant Disease Risk Assessment tool can be used^84^ (http://www.ircohe.net/IDRA).

It is always important to remind patients about the SPIC intervals and importance of home care, as in the absence of apparent complications, patients may gradually become less vigilant and committed.^17^ Also, it is of paramount importance to ensure that dental professionals receive comprehensive training and possess the necessary knowledge for conducting SPIC, as well as knowing when to refer patients to specialists.^86^

## Conclusion

A regular and structured SPIC programme is essential for the long-term maintenance of peri-implant tissue health:

- Preventive measures should commence before implant placement and persist throughout the patient's lifetime
- Key elements of a tailored SPIC programme should include monitoring of peri-implant tissue health and stability, oral health promotion and effective control and management of risk factors. Regular professional plaque biofilm removal stands as a crucial component
- SPIC should address not only patients with peri-implant health but also those diagnosed and treated for peri-implant disease.
